# Quantitative Evaluation of Carbon Fiber Dispersion in Amorphous Calcium Silicate Hydrate-Based Contact-Hardening Composites

**DOI:** 10.3390/molecules26030726

**Published:** 2021-01-30

**Authors:** Guangxiang Ji, Guangqi Xiong, Xiaoqin Peng, Shuping Wang, Chong Wang, Keke Sun, Lu Zeng

**Affiliations:** College of Materials Science and Engineering, Chongqing University, Chongqing 400045, China; jigx@cqu.edu.cn (G.J.); 20133270@cqu.edu.cn (G.X.); pxq01@cqu.edu.cn (X.P.); chongwang@cqu.edu.cn (C.W.); 20170901001@cqu.edu.cn (K.S.); zool@foxmail.com (L.Z.)

**Keywords:** contact-hardening composites, electrical conductivity, carbon fiber dispersion, digital image processing technology, Taipalu model

## Abstract

Carbon fiber dispersion has a substantial influence on the properties of amorphous calcium silicate hydrate-based contact-hardening composites. In this study, a mixture of carbon fiber and calcium silicate hydrate powder was compressed into solid composites at 40 MPa for one minute. The mechanical properties and electrical resistivity of the solid materials were measured, and the dispersion of carbon fibers was quantitatively evaluated by digital image processing technology. The Taipalu model was used to build the correlation between the electrical resistivity of the composites and the carbon fiber dispersion. The results of the electrical resistivity showed that the down threshold of carbon fiber content in the contact-hardening composites was 1.0 wt.% and the electrical resistivity was 30,000 Ω·cm. As the fiber content increased to 2.0 wt.%, the electrical resistivity dropped to 2550 Ω·cm, which was attributed to the increase in fiber dispersion uniformity in the solid composites, and the value of the fiber distribution coefficient reached a maximum value of 0.743. A subsequent decrease in the uniformity of the fiber dispersion was observed at a high fiber content. In addition, the carbon fiber content showed a slight influence on the fiber orientation in the contact-hardening composites.

## 1. Introduction

Several decades ago, researchers discovered that calcium silicate hydrate powder ((C-S-H), where C=CaO, S=SiO_2_ and H=H_2_O) in an amorphous nature can be directly compressed to produce water-resistant artificial blocks with sufficient strength [1,2,3,4]. This process is similar to powder metallurgy where the strength can be derived from the bonds resulting from the deformation of the particles when they are in contact under pressure. In this case, Glukhovsky and Pu et al. [5,6] postulated “contact-hardening cementitious properties” for unstable or metastable minerals, and amorphous calcium silicate hydrate was one of the available materials for producing solid blocks with water-resistant properties. This behavior is due to the decrease in high surface energy under compression, leading to the bridging between particles in contact or even recrystallization between the surfaces of the particles [2,5].

To date, several investigations have been conducted on the contact-hardening properties of amorphous calcium silicate hydrate powder, and it has been found that solid compacts with substantial strength can be obtained in a short period at room temperature; for instance, pressing for 1 to 10 min [7,8]. No further treatment, such as standard curing, heat curing or sintering, is required. The hardening process is somehow similar to the “cold sintering” process of ceramics where interparticulate bonding is formed [9]. This provides an innovative approach to prepare composites with other types of materials, such as metallurgy powder, inorganic substances and organic substances. Calcium silicate hydrate-based electrical conductive material was prepared when copper powder was incorporated [5]. A new type of bioceramic was prepared by compaction of weakly crystallized calcium phosphate and calcium silicate hydrates [10]. Udawatte et al. [11] used the hydrothermal hot pressing method to solidify calcium silicate hydrate powders to prepare artificial wood, where the reinforcement substance was chitosan, while the authors used the contact-hardening method to produce artificial wood from a mixture of amorphous calcium silicate hydrate powder, polypropylene fiber and latex [12].. This is because these materials are more easily dispersed in powdered materials than in slurries due to their surface chemical effect [13].

Compared to traditional materials, fiber-reinforced composites exhibit improved mechanical performance. Among many fiber types, carbon fiber is an advanced fiber material with a high strength and high modulus, and a carbon content higher than 95%. Moreover, carbon fiber has a high flexural strength, a low density, high specific performance, low creep, favorable fatigue resistance, and ultra-high temperature resistance in a non-oxidizing environment [14]. Therefore, in a previous study on contact-hardening composites, amorphous calcium silicate hydrate powders could be reinforced with carbon fibers to obtain special functions, such as electrical conductive materials [15]. In addition, the carbon reinforced cementitious composites have great potential for self-heating and de-icing applications in the construction field [16,17]. Considering that the dispersion of carbon fibers can have a substantial effect on the mechanical and electrical properties of the composites [17,18], it is necessary to investigate the influence of fiber dispersion on the contact-hardening materials. 

The accurate quantification of fiber distribution and orientation is the foundation of fiber dispersion characterization, and some direct and indirect test methods have been developed for fiber-reinforced cementitious materials and other types of composites. Indirect test methods, such as AC-impedance spectroscopy [19], X-ray computer tomography [20], magnetic monitoring [21], and translucent fluid [22] were mainly used to evaluate the conductivity of composites. As for the direct test method, digital image processing technology (DIPT) has been widely applied for the fiber reinforced composites in recent years [23,24]. Cao et al. [25] determined the relationship between the fiber distribution and properties of fiber-reinforced cement-based composites (FRCs) by using DIPT. Lataste et al. [26] used DIPT to characterize the distribution of steel fibers in concrete with superhigh fiber contents and proposed the concept of “intensity of orientation of fibers”. Kim et al. [13,27,28] collected fluorescence images of polyvinyl alcohol (PVA) fibers in cementitious composites by using a charge coupled device (CCD) digital camera with a fluorescence microscope and evaluated fiber dispersion on the basis of differences in the number of fibers per unit area. However, there is a lack of published papers addressing the study of carbon fiber distribution and orientation in amorphous calcium silicate hydrate contact-hardening composites. Thus, how the fiber content affects the mechanical properties and electrical resistivity of contact-hardening composites is still unclear.

This study aims to investigate the relationship between the distribution of carbon fiber content and the electrical resistivity of a calcium silicate hydrate-based contact-hardening composite. The mechanical properties and electrical resistivity of the composites with different carbon fiber contents were measured. DIPT was used to analyze the fiber distribution and orientation characteristics of the composites with different carbon fiber content. Moreover, the modified Taipalu model was performed to establish the relationship between the fiber dispersion and electrical resistivity. These results are of significance for the design, preparation, and property optimization of contact-hardening fiber composites.

## 2. Raw Materials and Test Methods

### 2.1. Raw Materials

**Figure 1:**
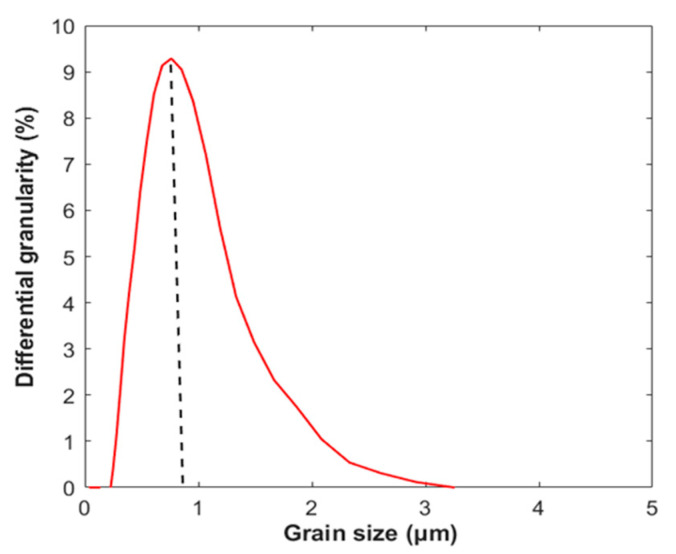
Particle size distribution of calcium silicate hydrate powder.

The calcium silicate hydrate powder used in this study was synthesized by a dynamical hydrothermal method from a mixture of calcium oxide (CaO) and siliceous materials (SiO_2_). Calcium oxide (with a free CaO content of 88.8%) was supplied by the Shandong lime plant in Chongqing. Its Blaine fineness was 410 m^2^/kg, and the average particle size was 0.8 μm. Quartz powder (with SiO_2_ content of 96.5%) was milled for 90 min from quartz sand (supplied by Hunan Magnificent Vision New Materials Technology Co. Ltd.). The materials were dried at 105 °C to remove absorbed water before hydrothermal synthesis. Calcium oxide, quartz powder and water were mixed in an autoclave (20 L, Weihai Chemical Machinery Co. Ltd., Weihai, China). The Ca/Si molar ratio of the starting materials was 1:1, and the water/solid weight ratio was 5:1. It took 5 h for the temperature to increase from room temperature to 180 °C, and it was then kept constant at 180 °C for 3 h. The mixture was stirred at a rate of approximately 400 r/min. When the reaction was finished, the mixture was quickly cooled to 60 °C before being filtered. The filtered product was oven-dried at 60 °C for approximately 24 h according to the previous study [29], and the moisture content of the powder used in this study was 11.75%, compared to the powder supercritical dried at 105 °C to the constant weight. The oven-dried powder with moisture content of 11.75% was then dispersed into powders for a duration of 10 s by a mixer. Laser granulometry supplied by Haixinrui Technology Co., Ltd. (Beijing, China) was used to evaluate the particle size of the calcium silicate hydrate powders described above. The particle size distribution curve is shown in Figure 1. The average size of the particles was approximately 1 μm.

**Figure 2:**
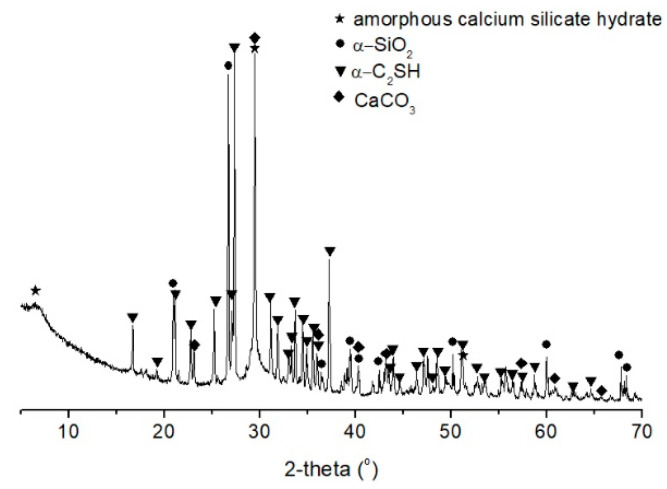
XRD pattern of synthesized calcium silicate hydrate powder.

The mineral phases of the synthesized calcium silicate hydrate powder were determined by using X-ray diffraction (XRD, RigakuD/max-1200, Cu Kα, Osaka, Japan) with an output power of 3 kW. A step size of 0.02°, scanning rate of 2°/min and scanning range from 15° to 65° were applied. The XRD pattern of the powders is shown in Figure 2. The diffused peaks can be observed at d-spacings of approximately 1.4 nm (2θ of 6.5°), 0.303 nm (2θ of 29.4°) and 0.182 nm (2θ of 51.2°), which are typical for the amorphous calcium silicate hydrate [30]. In addition, crystalline phases, including α-SiO_2_, α-C_2_SH and calcite, were also detected in the powder. α-SiO_2_ with d-spacings of 0.425, 0.334, 0.1819 and 0.1541 nm was derived from the quartz powder. α-C_2_SH is a crystalline calcium silicate hydrate with d-spacings of 0.421, 0.324, 0.277, 0.250, 0.218, 0.179 nm [31]. The presence of calcite was due to the carbonation of calcium silicate hydrate during drying and storage process. 

**Figure 3:**
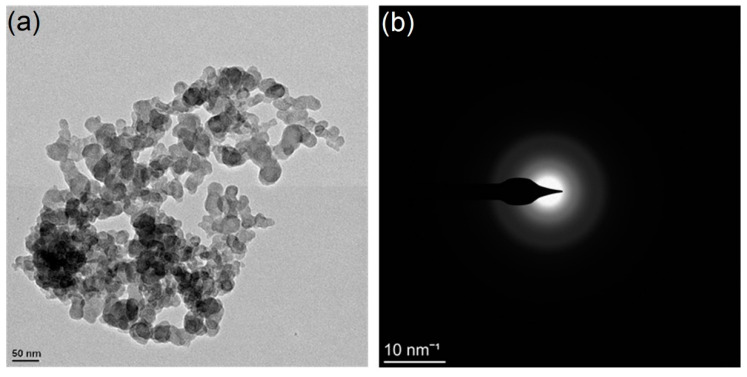
TEM microscopy of amorphous calcium silicate hydrate: (**a**) morphology of amorphous calcium silicate hydrate; (**b**) selected area electron diffraction (SAED) pattern.

The nanostructure of the synthetic powder was characterized by transmission electron microscopy (TEM, Libra 200, Zeiss, Germany) with a maximum resolution of 1.4 nm. The particles consisted of globular particles with sizes of approximately 20 to 50 nm (Figure 3a), and the selected area electron diffraction (SAED) pattern in Figure 3b further demonstrated the presence of amorphous calcium silicate hydrate due to the presence of a faint halo around it.

The carbon fibers in the current study were supplied by Zhongfu Shenying Carbon Fiber Co., Ltd. The properties are shown in Table 1.

### 2.2. Preparation of Contact-Hardening Composites

**Figure 4:**
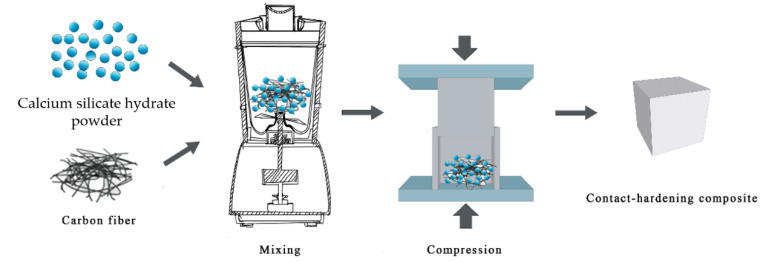
Flow chart of the preparation of the contact-hardening composites.

In this study, a mixer (O-500Y, Oubo high speed pulverizer, Jinhua, China) was used to mix calcium silicate hydrate powders and carbon fibers at different weight ratios for approximately 10 s to produce a homogeneous mixture before being poured into a 40 × 100 × 160 mm^3^ steel mold. A certain amount of the mixture was compressed by increasing the pressure on both sides with a compression pressure of 40 MPa at a rate of 0.4 MPa/s. The pressure was maintained at 40 MPa for 1 min. The contact-hardening specimen with size of 40 × 40 × 160 mm^3^ prepared from the mixture of calcium silicate hydrate and carbon fiber was removed from the mold immediately after being compacted. The procedure of the contact-hardening composite preparation is shown in the flow chart below (Figure 4). More details of the preparation of the hardening specimens were described in [32]. The influence of fiber content (ranging from 0.5 wt.% to 4.0 wt.%) on the properties of the contact-hardening composite was investigated.

### 2.3. Characterization of the Contact-Hardening Composites

The flexural strength, compressive strength, and bulk density of the specimens were measured according to the *Test Methods for Concrete Blocks and Bricks* (GB/T 4111-2013).

The electrical conductivity was tested according to the two-point electrical conductance test principle by using the inductance capacitance resistance (LCR) method [33]. The specimen was placed between two metal plates. Wet cotton cloths were placed between the specimen and metal plates to ensure that they were in full contact. When the test was carried out, an LCR bridge was used to alternate the current with a frequency setting of 1 Hz to reduce the influence of the direct current and low frequency on the polarization of the specimen. The test was conducted after 2 min of contact, and steady-state of the current was achieved. The electrical resistivity ρ (Ω·cm) was calculated by using Equation (1).

(1) $$\rho =\frac{RS}{l}$$

where *R* (Ω) is the electrical resistance of the specimen, *S* (cm^2^) is the cross-section area of the specimen (cm^2^) and *l* (cm) is the length of the specimen.

### 2.4. Image Method for Fiber Distribution and Orientation

To improve the accuracy and validity of the calculation results of carbon fiber distribution and orientation, three specimens were prepared in each mixing proportion, and each specimen was cut into 5 pieces. Initially, the surface of the cross-section was polished roughly on an electrical grinding machine by using ^#^300 coarse sandpaper. The specimens were then polished by using finer sandpapers, i.e., ^#^600 paper, ^#^1000 paper and then ^#^1500 fine sandpaper, to produce an adequately smooth surface with minimum damage. A high-resolution charge-coupled device camera (CCD, EOS 80D) supplied by Canon was used to take photographs of the polished surface.

**Figure 5:**
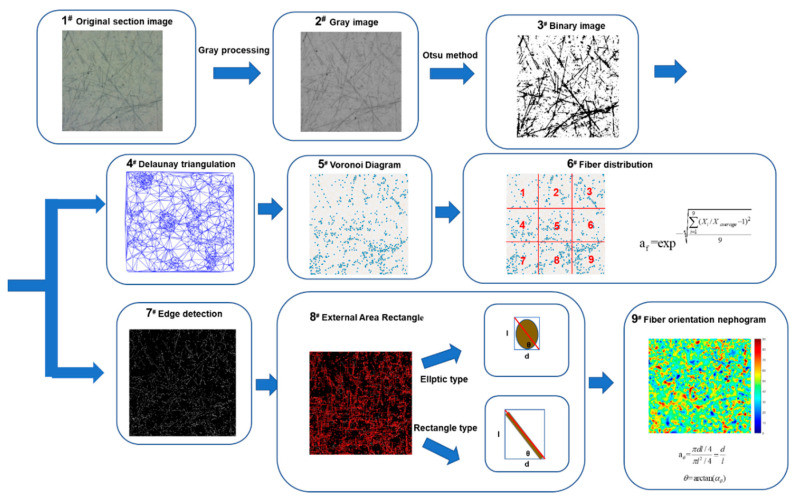
Flow chart of the digital image processing procedure.

Digital image processing technology (DIPT) was used to quantify the distribution and orientation of the carbon fibers in this study. The whole process is shown in Figure 5. The original image was converted into a grey image. It was then converted into a binary image by referring to the maximum interclass variance method proposed by Otsu [34], and thus, the geometric morphological information about carbon fibers was obtained, as shown in Figure 5-3^#^.

In terms of the distribution of the carbon fibers, the centroids of each carbon fiber were extracted by using the “Centroid Function” in the MATLAB 2018a software. The Voronoi diagram of the distribution of the centroids (see Figure 5-5^#^) was generated based on the Delaunay triangulation method (see Figure 5-4^#^). The specific description of the Delaunay triangulation method and Voronoi diagram can be found in Bosman’s study [35].

**Figure 6:**
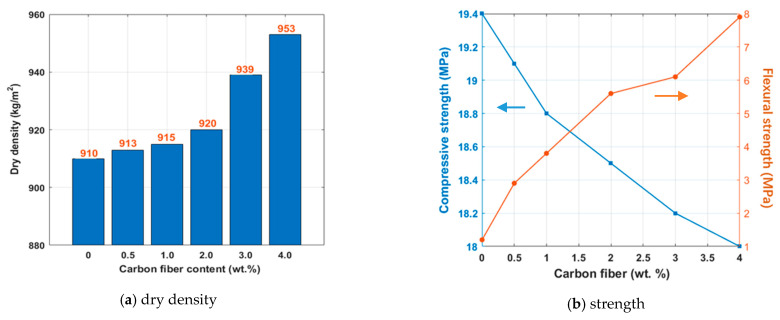
Physical properties of the contact-hardening composites with different carbon fiber contents: (**a**) dry density; (**b**) compressive and flexural strength.

**Figure 7:**
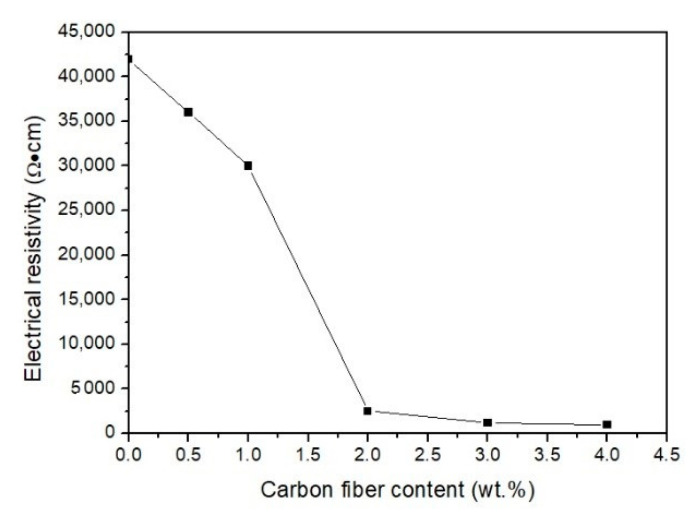
Electrical resistivity of the composites with different carbon fiber contents.

**Figure 8:**
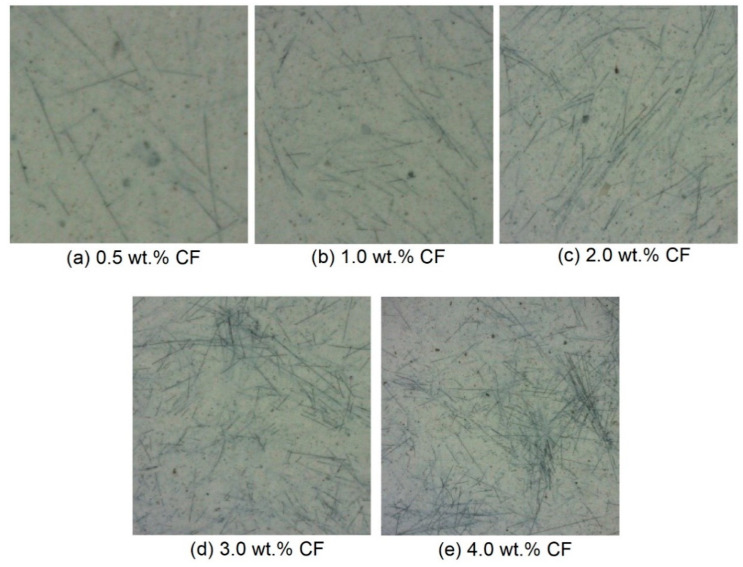
Original images of carbon fibers in the contact-hardening composites with different fiber contents: (**a**) 0.5 wt.%; (**b**) 1.0 wt.%; (**c**) 2.0 wt.%; (**d**) 3.0 wt.%; (**e**) 4.0 wt.%.

With regard to the orientation of the carbon fibers, the Sobel operator [36] was used to verify the edge profile of the carbon fiber in a binary image. The edge contour of the fibers was further quantified by using the “External Area Rectangle” in MATLAB. The orientation of the carbon fibers at the cross-section was presented in two ways. When cutting along the cross-section, fibers showed elliptical features and when cut along the longitudinal section, the fibers showed rectangular features, as shown in Figure 5-8^#^. The orientation of the carbon fibers was calculated based on the study conducted by Song [37], and the colormap in MATLAB was used to characterize the fiber orientation nephogram. 

## 3. Results 

### 3.1. Mechanical Properties of Contact-Hardening Composites

The compressive strength, flexural strength, and bulk density of the contact-hardening composites are shown in Figure 6.

From Figure 6a, it can be seen that as the carbon fiber content increased from 0 to 4.0 wt.%, the apparent density gradually increased since the density of the carbon fibers was higher than that of the amorphous calcium silicate hydrate-based contact-hardening specimen prepared at 40 MPa. The structure became more compact with an increase in fiber content. An opposite tendency was observed in the compressive strength of the contact-hardening composites, in which it decreased slightly from 19.4 MPa to 18.0 MPa as the fiber content increased to 4.0 wt.% (Figure 6b). This is probably because calcium silicate hydrate powders were not able to cover the surface of the carbon fibers, and it is easy to form defects at the interface between fiber and C-S-H. With increasing carbon fiber content, the number of defects increased further. During compression, the microcracks expanded more rapidly and therefore caused failure to occur earlier. However, the flexural strength was greatly improved by increasing the carbon fiber content. When the carbon fiber contents were 0.5, 1.0, 2.0, 3.0, and 4.0 wt.%, the flexural strength increased by 141, 216, 367, 408, and 558%, respectively, compared with the specimen that contained no carbon fibers. This can be explained by the following reason. The flexural strength is the ability of a material to resist bending without breaking. The effective bearing area of the carbon fibers increased with increasing fiber numbers, improving the brittleness of the matrix, as described by Jia et al. [38].

### 3.2. Electrical Resistivity of Contact-Hardening Composites

Resistivity is an important physical parameter for measuring the electrical conductivity of engineering materials, especially fiber-reinforced cement-based materials. The influence of the carbon fiber content on the electrical resistivity of contact-hardening composites is shown in Figure 7.

From Figure 7, it can be seen that the electrical resistivity of the contact-hardening composites decreased from 42,000 Ω·cm to 30,000 Ω·cm as the carbon fiber content increased from 0 to 1.0 wt.%, but a more dramatic decrease occurred at fiber contents between 1.0 wt.% and 2.0 wt.%. This was due to the percolation phenomenon that when the conductive phase content in the nonconductive matrix exceeded a certain threshold, the conductive phases would connect with each other and lead to a sudden change in electrical resistivity [39]. The mass fraction of conductive materials where the electrical resistivity dramatically decreased with increasing carbon fiber content is called the down threshold, which was 1 wt.% for the material studied with an electrical resistivity of 30,000 Ω·cm. This was followed by a dramatic decrease in electrical resistivity of 2550 Ω·cm at a fiber content of 2.0 wt.%, which was considered the upper threshold in this study.

In addition, when the carbon fiber content was lower than 1.0 wt.%, the value of electrical resistivity was relatively high due to the comparatively large distance between the carbon fibers. In this case, the carbon fibers failed to connect with each other to form a network. Therefore, a good electrical path was unlikely formed to provide considerable conductance. As the carbon fiber content increased, the distance between carbon fibers decreased, the number of connections increased and a network structure formed. Electrical electricity was conducted by the tunneling effect of the carbon fibers, as demonstrated by Zhang et al. [40]. In addition, the electrical resistivity decreased dramatically at fiber contents of between 1.0 wt.% and 2.0 wt.%. When the carbon fiber content was higher than 2.0 wt.%, the electrical resistivity started to slow down. This was because the conductive network of carbon fibers was already formed and an excess of carbon fiber content would probably increase the number of fiber clusters, leading to decreasing uniformity of the fiber distribution in the matrix. As a result, only a minor change was observed in the electrical resistivity. More information about the fiber distribution is discussed in the following section.

According to the above results, an optimum dispersion of carbon fibers in contact-hardening composites appeared at a carbon fiber content of 2.0 wt.%.

### 3.3. Image Analysis

#### 3.3.1. Analysis of the Carbon Fiber Distribution

Carbon fibers in the contact-hardening composite can connect with each other and become electrical conductors. In addition, the distribution of the fibers was also assumed to play an important role in inhibiting the propagation of cracks and improving the flexural strength of the specimen.

**Figure 9:**
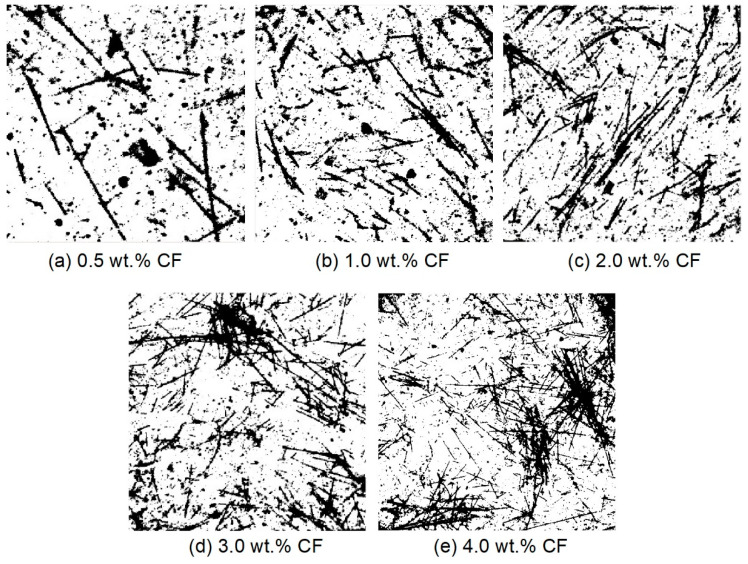
Binary images of the carbon fibers after image processing from Figure 8 with different fiber contents: (**a**) 0.5 wt.%; (**b**) 1.0 wt.%; (**c**) 2.0 wt.%; (**d**) 3.0 wt.%; (**e**) 4.0 wt.%.

Figure 8 shows the original high-resolution CCD images of the cross-section of the contact-hardening composites with different carbon fiber contents, and Figure 9 presents the binary images from Figure 8 after processing by using Otsu’s method [34].

**Figure 10:**
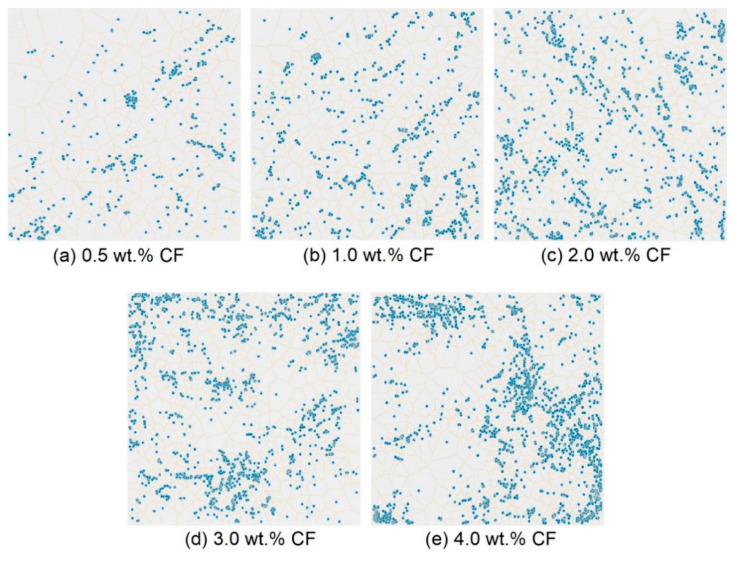
Voronoi diagram of the carbon fiber distribution under different carbon fiber contents: (**a**) 0.5 wt.%; (**b**) 1.0 wt.%; (**c**) 2.0 wt.%; (**d**) 3.0 wt.%; (**e**) 4.0 wt.%.

**Figure 11:**
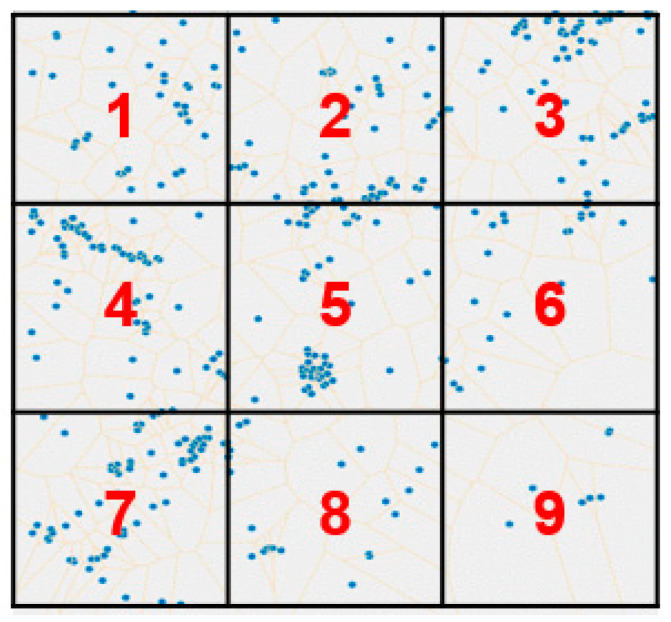
Subsection of the Voronoi diagram.

Voronoi diagrams with different carbon fiber contents are shown in Figure 10. Each picture was divided into nine subsections, which are shown in Figure 11. The number of fibers in each subsection was counted. The formula of the fiber distribution coefficient (*α_f_*) proposed by Han et al. [41] was used to quantitatively evaluate the uniformity of the fiber distribution, as described in Equation (2).

(2) $$\alpha_{f}=\frac{1}{15}\overset{15}{\underset{j=1}{\sum }}\exp^{—\sqrt{\frac{\sum_{i=1}^{n}{\left(X_{i}/X_{average}-1\right)}^{2}}{n}}}$$

where *n* = 9, which reflects the number of segmented portions, *X_i_* is the number of carbon fibers positioned in the *i*th area, and *X_average_* is the average number of carbon fibers in all areas. α*_f_* is the fiber distribution coefficient. α*_f_* = 1 indicates that fibers are completely homogeneously distributed, and α*_f_* = 0 indicates that the agglomeration of fibers. j is the number of the cross-section. 

**Figure 12:**
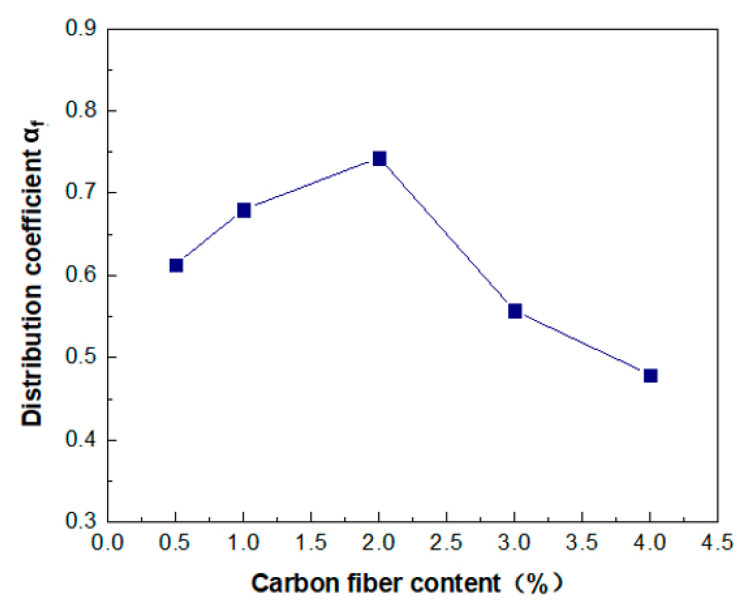
Fiber distribution coefficient *α_f_* of the composites with different carbon fiber contents.

For each mixture, a section that was close to the average carbon fiber distribution coefficient was selected from 15 sections of each mixing proportion, and the section comprehensively reflected the dispersion characteristics of fibers with different mass fractions in the composites. The statistic of the number of carbon fibers for the different additions are shown in Table 2. The calculation results are shown in Figure 12. It can be seen that with the increase in carbon fiber content, the values of α*_f_* first increased and then decreased. The values were 0.613, 0.680, 0.743, 0.557 and 0.479, corresponding to the fiber content of 0.5, 1.0, 2.0, 3.0 and 4.0 wt.%, respectively. At a fiber content of 0.5 wt.%, the number of fibers was not enough to be dispersed in the whole area of the matrix. The initial increase in the value of *α_f_* with an increase in fiber content was ascribed to the decrease in the discreteness degree of the fibers in each subarea as the fibers gradually came into contact. As a result, the distribution of the fibers became more homogeneous in the matrix as the fiber content increased up to 2.0 wt.%, and *α_f_* increased to 0.743. The subsequent decrease in the *α_f_* value when the fiber content was beyond 2 wt.%, demonstrating that a cluster of fibers occurred because of the excessive amount of fibers. Such a result was consistent with Kang’s research [28]. This demonstrates that a carbon fiber content of 2 wt.% was substantial in the amorphous calcium silicate hydrate-based contact-hardening composite, which also provided an optimum value of electrical resistivity and rapid growth in flexural strength.

#### 3.3.2. Analysis of the Fiber Orientation

**Figure 13:**
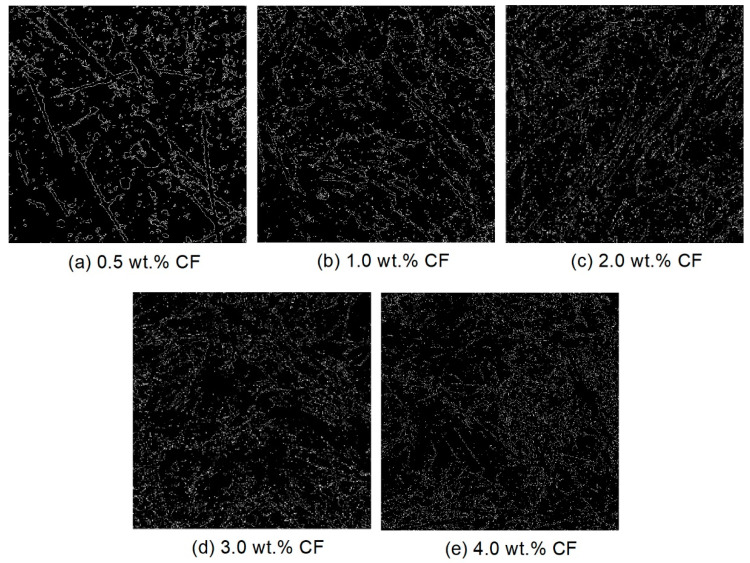
Sobel operator contour extraction of the carbon fibers of the contact-hardening composites with different contents: (**a**) 0.5 wt.%; (**b**) 1.0 wt.%; (**c**) 2.0 wt.%; (**d**) 3.0 wt.%; (**e**) 4.0 wt.%.

**Figure 14:**
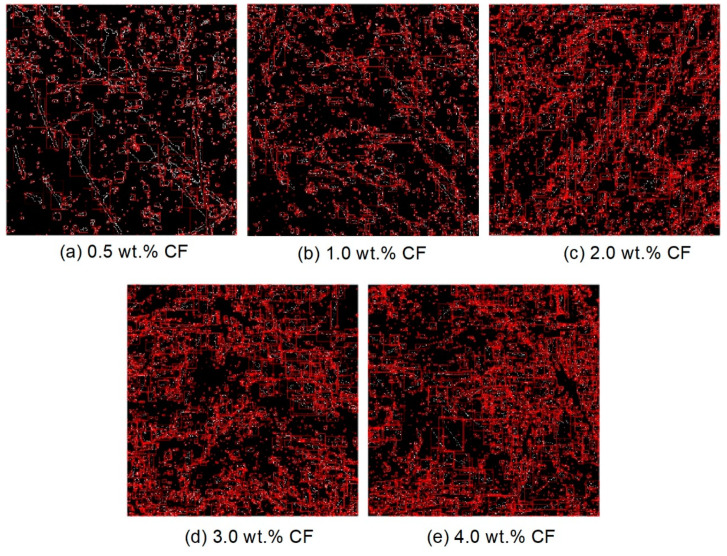
Fiber orientation binary image of the contact-hardening composites with different fiber contents using an external area rectangle (EAR): (**a**) 0.5 wt.%; (**b**) 1.0 wt.%; (**c**) 2.0 wt.%; (**d**) 3.0 wt.%; (**e**) 4.0 wt.%.

The orientation of the carbon fibers provided a clear and easy way to characterize the carrying capacity. When conducting an analysis of the orientation, the image processing procedures described in Section 2.4 were used (see Figure 5-7^#^–9^#^). The “Sobel operator” of the MATLAB 2018-a software, which is a typical edge detection operator based on the first-order derivative of the image brightness function, was used in the current study. The algorithm is more applicable than the Prewitt operator and Roberts operator because it introduces local average operator [42]. The extraction figure is shown in Figure 13. Then, the external area rectangle (EAR) was used to measure the fiber orientation, which is shown in Figure 14. When using the EAR, each fiber was wrapped by drawing the external area rectangle.

The inclined angle coefficient *θ* of these two types of sections of carbon fibers can be calculated by Equation (3) [37]. Finally, the fiber orientation angle nephogram was used to illustrate the orientation of the carbon fibers, which is shown in Figure 5-9#.

(3) $$\theta =\arctan\frac{l_{r}}{d}$$

where *l_r_* is the length of the external area rectangle and *d* is the width of the external area rectangle. *θ* tending to 90° indicates that the carbon fibers are aligned vertically, showing a weak carrying capacity, and *θ* = 0° means that the carbon fibers are aligned laterally, showing a strong carrying capacity.

**Figure 15:**
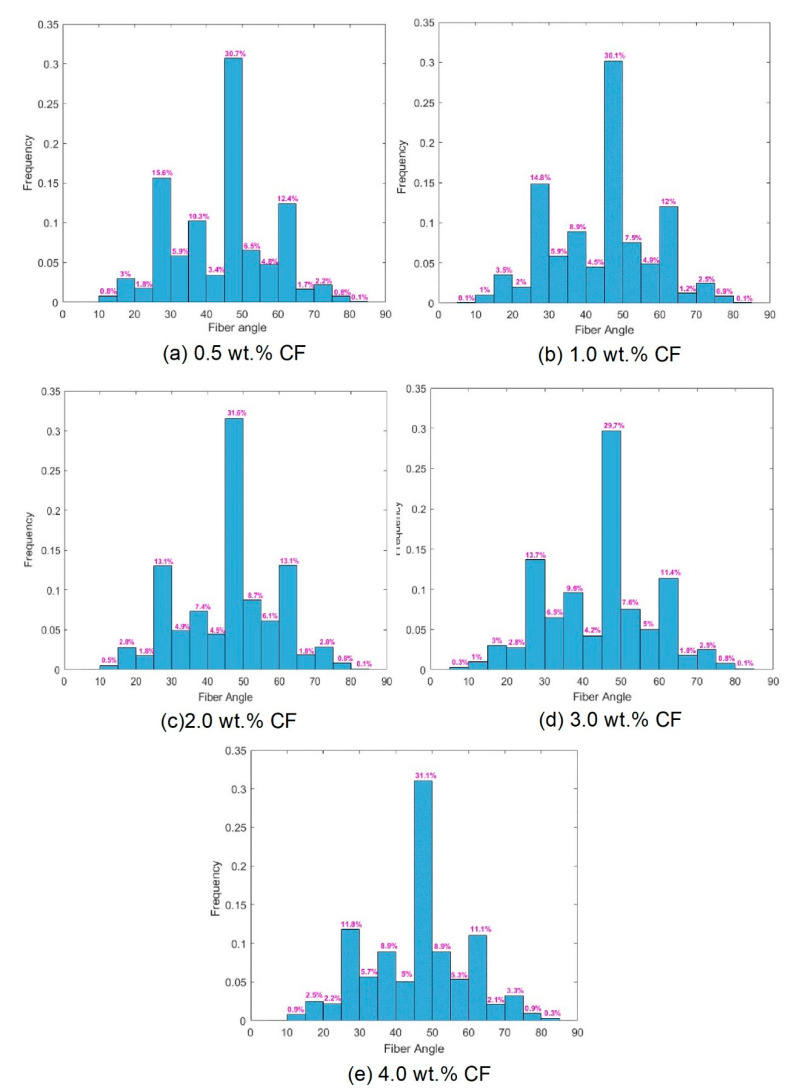
Histogram of the orientation angles of the carbon fibers with different contents in the contact-hardening composites: (**a**) 0.5 wt.%; (**b**) 1.0 wt.%; (**c**) 2.0 wt.%; (**d**) 3.0 wt.%; (**e**) 4.0 wt.%.

The carbon fiber orientation angle was further divided into every five degrees to calculate the ratio of the number of carbon fibers at that angle range to the total number of fibers in each section. The histograms of the carbon fiber orientation angles are shown in Figure 15.

**Figure 16:**
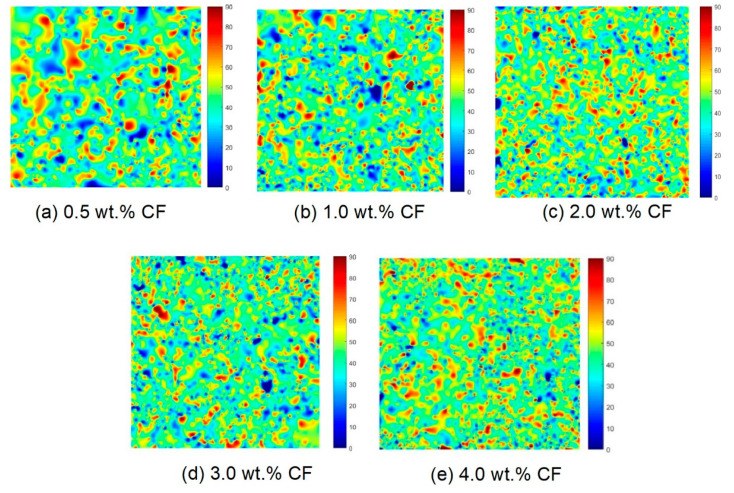
Nephogram of the orientation angles of the carbon fibers (Unit: °) in the contact-hardening composites with different carbon fiber contents: (**a**) 0.5 wt.%; (**b**) 1.0 wt.%; (**c**) 2.0 wt.%; (**d**) 3.0 wt.%; (**e**) 4.0 wt.%.

From Figure 15, it can be seen that the carbon fibers in amorphous calcium silicate hydrate-based contact-hardening composites were randomly distributed. The fiber orientation angles in each group mainly ranged from 45° to 50°. Approximately 30.7, 30.1, 31.6, 29.7 and 31.1% of the fibers were in this angle range for the composites containing carbon fiber contents of 0.5, 1.0, 2.0, 3.0 and 4.0 wt.%, respectively. Meanwhile, 89.6, 88.6, 89.4, 87.7 and 87.8% of the fibers were in the angle range of between 25° and 65°, which showed no obvious difference. This result indicates that fibers mainly oriented in the angle range of 40° to 50°. The fiber content has little influence on the fiber orientation of contact-hardening composites. In traditional cement paste, the fiber orientation increases gradually with the increase in viscosity and yield stress [43]. However, in the process of powder compression, the orientation of carbon fiber is only affected by the compression pressure, so the increase in fiber content has little influence on the orientation. The figure suggests that with increasing fiber content, the frequency of connection between fibers in each subarea increased, although no substantial changes in the fiber orientation angles were observed in the fiber distribution (Figure 16). However, an excessive fiber content (e.g., 4.0 wt.% in Figure 16e) increases the possibility of fibers connecting, winding, and balling during the contact-hardening process, and the orientation angle becomes increasingly random.

## 4. Discussion

### 4.1. Correlation between the Electrical Resistivity and Dispersion Parameters

#### 4.1.1. The Taipalus Model

The electrical resistivity reflects the electrical conductance properties of contact-hardening composites in different fiber contents, but it fails to intuitively reflect the real characteristics of carbon fiber dispersion. It is necessary to establish the relationship model between the electrical resistivity and dispersion. Based on the thermal and electrical conductive model of particles’ filler composites [44], Weber and Kamal [45] proposed the fiber contact model to calculate the volume resistivity of FRC (fiber-reinforced composites). Taipalus et al. [46] improved Weber and Kamal’s model by introducing the electrical resistivity of the matrix. The modified classical model has been widely used to study the electrical effect of carbon fiber on polypropylene [47], rod-filled composites [48], and hexagonal-boron nitride/methyl-vinyl-silicone rubber (h-BN/MVQ) [49].These theoretical values of resistivity are somehow dependent on the experimental results. However, there is a lack of studies on the classical model that has been conducted on contact-hardening composites. Therefore, it is necessary to investigate the carbon fiber dispersion of the novel constituent system in the framework by applying the Taipalus model. The electrical resistivity of the composite can be defined by Equation (4).

(4) $$\frac{1}{\rho_{c}}=\frac{1}{\rho_{m}}+\frac{4d_{c}l_{0}cos^{2}\theta V_{p}\sigma_{f}X}{\pi d_{0}{}^{2}}$$

where $\rho_{c}$ in Ω·cm is the electrical resistivity of the composites, $\rho_{m}$ in Ω·cm is the electrical resistivity of the matrix, and $d_{c}$ is the diameter of the contact circle, the value of which is 40 μm according to Ref. [50]. $d_{0}$ is the diameter of the carbon fiber with a value of 40 μm, and $l_{0}$ is the average carbon fiber length, which equals 10 mm. $\sigma_{f}$ is the electrical resistivity of the carbon fibers, and the value is 290 Ω·cm. *θ*, $V_{p}$ and X are explained below.

*θ* denotes the orientation angle of the carbon fibers, which can be calculated by Equation (5).

(5) $$\theta =\int_{0}^{\frac{\pi }{2}}\rho \left(\theta \right)d\theta =\overset{n}{\underset{i=1}{\sum }}\rho \left(\theta_{i}\right)\theta_{i}$$

where ρ(θ) is the probability function of the carbon fiber orientation, *n* is the step length of the differential equation, and *n* = 9 in this study. $\theta_{i}$ is the fiber orientation angle of the *i^th^* subsection, which is defined as the average of the interval between the minimum and maximum angles. $\rho \left(\theta_{i}\right)$ is the probability value of $\theta_{i}$.

The parameter $V_{p}$ denotes the volume fraction of carbon fibers involved in forming conductive paths, which can be expressed by Equation (6) [46].

(6) $$V_{p}=\{\begin{array}{c} 0\;V_{f}\leq V_{pet}\\ \frac{V_{t}-V_{per}}{V_{f}-V_{per}}V_{t}\;V_{per}<V_{f}\leq V_{t}\\ v_{t}\;V_{t}<V_{f} \end{array}\;$$

where $V_{f}$ is the mass fraction of carbon fibers in the composites. $V_{per}$ is the threshold mass fraction corresponding to the lower threshold, which means that there are not enough carbon fibers to bridge each other to form pathways, and $V_{per}$ was 1.0 wt. % in this study. $V_{t}$ is the mass fraction corresponding to the upper threshold, which means that a sufficient amount of carbon fibers can bridge into pathways so that the electrical resistivity of the composite does not increase obviously, and $V_{t}$ was 2.0 wt.% in this research.

The parameter *X* denotes the contact factor of the carbon fibers per unit volume. Weber and Kamal [45] provided an empirical formula to establish *X* and the contact numbers of the fibers. However, due to the difficulty of determining the maximum numbers of contacts, this formula cannot be universally applied to other complicated systems. Therefore, in this study, a modified formula is provided in Equation (7).

(7) $$X=\frac{k\left(1-\alpha_{f}\right)+b}{100V_{t}}$$

where $\alpha_{f}$ is the fiber distribution coefficient. When the carbon fibers were evenly distributed, $\alpha_{f}$ was 1. In this case, each fiber can be regarded as being completely covered by calcium silicate hydrate powder without direct contact, and as a result, *X* = 0. $\alpha_{f}$ was zero when the carbon fibers were completely agglomerated. They may be considered to be in contact with each other.

By substituting Equations (5)–(7) into the Equation (4), the empirical formula of the carbon fiber distribution can be obtained, as shown in Equation (8).

(8) $$\frac{2500\pi d_{0}{}^{2}}{d_{c}l_{o}cos^{2}\theta \sigma_{f}}\left(\frac{1}{\rho_{c}}-\frac{1}{\rho_{m}}\right)=\left[k\left(1-\alpha_{f}\right)+b\right]$$

The residual can be calculated based on Equation (9)

(9) $$\mathrm{Residual}=\left|\rho_{model}-\rho_{experiment}\right|$$

where $\rho_{model}$ is the predictive electrical resistivity, and $\rho_{experiment}$ is the experimental electrical resistivity.

#### 4.1.2. Model Validation and Analysis

To validate the Taipalu model, all the parameters involved in Equations (4)–(7) as well as R-square (*R*^2^), were solved by the software MATLAB 2018-a and listed in Table 3.

From Table 3, the R-square value is 0.976, which shows that there is obvious agreement between the predicted value from the model and the experimental results [51]. The parameter *V_p_* indicates that the electrical resistivity of the contact-hardening composites satisfies the percolation–threshold theory, as demonstrated in [39]. Moreover, *θ* has no substantial influence on increasing carbon fiber content due to the fibers bridging each other and the phenomenon of preferred orientation, which is completely different from the study of carbon-filled polymer composites reported in [45]. Moreover, when the carbon fiber content was lower than 2.0 wt.%, the distribution of carbon fibers had no direct effect on the conductivity of the composites due to the lack of adequate electrical paths in the composites. When the carbon fiber content was higher than 2.0 wt.%, the *cos*^2^*θ* was between 0.486 and 0.508, and it could be assumed that all the carbon fiber groups had a *cos*^2^*θ* value equal to 0.50. Substituting the parameters in Table 3 into Equation (8), the distribution coefficient $\alpha_{f}$ for the carbon fiber content of 2.0, 3.0 and 4.0 wt.% shown in Figure 14 can be described by Equation (10).

(10) $$\alpha_{f}=0.9236-\frac{448.0652}{\rho_{c}}$$

**Figure 17:**
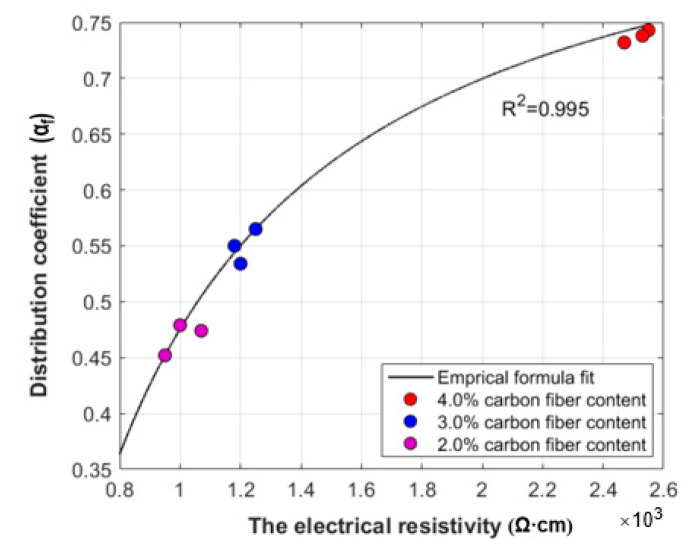
Relationship between the distribution $\alpha_{f}$ and the electrical resistivity $\rho_{c}$.

Figure 17 shows the relationship between $\alpha_{f}$ and the electrical resistivity. A strong relationship between $\alpha_{f}$ and *ρ*_c_ can be observed in the figure, and the electrical resistivity of the contract-hardening composites decreased with an increased carbon fiber content at a content higher than 2.0 wt.%, although a poorer fiber distribution appeared. This may be because the high carbon fiber content forms more conductive paths than low fiber dosages.

### 4.2. Correlation between Mechanical Properties and Dispersion Parameters

In Figure 6b, it shows that the compressive strength decreased with carbon fiber content. For the compressive strength, it is greatly affected by the interface between calcium silicate hydrate powder and carbon fibers. At a higher carbon fiber content, considering the complexity of carbon fiber agglomeration and the wall effect of powder, it is not easy to establish a relationship between fiber dispersion (including fiber distribution and orientation) and compressive strength. 

With respect to the flexural strength, the value increased with carbon fiber content (Figure 6b). However, it is shown in Figure 12 that the carbon fiber distribution coefficient of the contact-hardening composites increased first with increasing fiber content to 2 wt.%, and then decreased with an excessive fiber content. Meanwhile, the orientation angle ranges were not affected by the carbon fiber content as shown in Figure 15, due to the constant compression pressure applied on the specimen for one minute. Thus, there should be no direct relationship between flexural strength and carbon fiber distribution and orientation. For further study on the fiber distribution, however, Bosman et al. [35] proposed an indirect relationship between the flexural strength and fiber distribution. Gao et al. [17] investigated the influence of the spatial distribution coefficient on the flexural strength and toughness of carbon fiber-reinforced cement-based composites by the grey entropy correlation analysis. In this study, the product of the total number of the fibers in the composites and the distribution coefficient was termed as the effective carbon fiber numbers ($N_{e})$, as shown in Equation (11):

(11) $$N_{e}=\alpha_{f}X_{total}$$

where *N_e_* is the number of effective carbon fibers, *α_f_* is the distribution coefficient of carbon fibers, *X_total_* is the total number of carbon fibers. 

**Figure 18:**
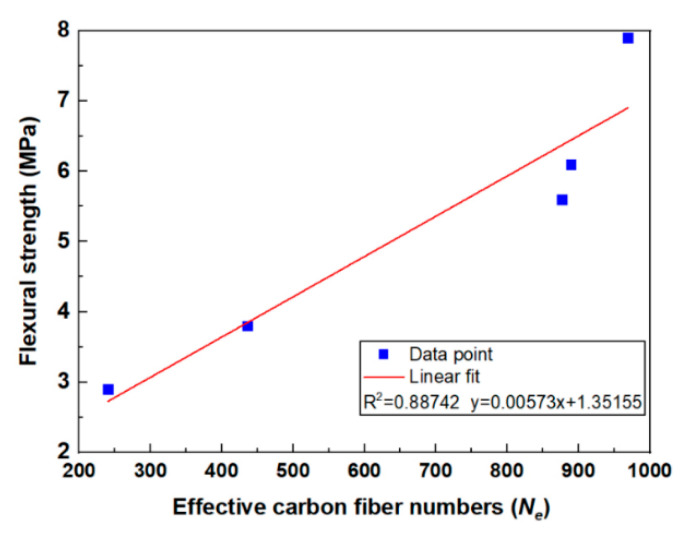
Relationship between the flexural strength of the composites and effective carbon fiber numbers.

The relationship between the flexural strength and the effective carbon fiber numbers is shown in Figure 18. In general, a linear relationship with R-square of 0.8872 was observed, and the relationship between flexural strength and the carbon fiber distribution coefficient can be further estimated by the following equation:

(12) $$\sigma_{cft}=\frac{573\alpha_{f}X_{total}+135,155}{10,000}$$

where $\sigma_{cft}$ is the flexural strength of the carbon fiber-reinforced contact-hardening composites. 

Despite the fact that the equation indicates that the distribution coefficient of carbon fiber affects the flexural properties of the matrix, it should also be noted that at a higher carbon fiber content, the effective carbon fiber numbers started with a slight change, but a remarked increase in flexural strength was still observed. This is due to the stack of the fiber observed by optical microscopy at a higher content. In future work, X-ray computed tomography (X-CT) will be used to characterize the spatial fiber in the composites to provide a three-dimensional fiber distribution prediction. 

## 5. Conclusions

(1) The carbon fiber content has a slight influence on the compressive strength and bulk density of amorphous calcium silicate hydrate-based contact-hardening composites, but it can greatly improve the flexural strength of the composites. When the carbon fiber content is 0.5, 1.0, 2.0, 3.0, and 4.0 wt.%, the flexural strength increases by 141, 216, 367, 408, and 558%, respectively, compared to the contact-hardening composite without fibers.

(2) The electrical resistivity of the specimens decreased with increasing carbon fiber content. A lower threshold for contact-hardening composites with carbon fibers appeared at a fiber content of 1.0 wt.%, and the electrical resistivity was 30,000 Ω·cm. A higher threshold was present at a fiber content of 2.0 wt.% with an electrical resistivity of 2550 Ω·cm. It was found that a carbon fiber content of 2.0 wt.% in the contact-hardening composite provided optimum uniformity of the carbon fiber dispersion in the contact-hardening composites.

(3) Direct analysis of the fiber distribution and orientation by using the imaging method suggests that by increasing the fiber content up to 2.0 wt.%, the value of the fiber distribution coefficient ($\alpha_{f}$) increased dramatically to 0.743, representing a more homogeneous distribution of fibers in the composite. When the fiber content was beyond 2 wt.%, the value of $\alpha_{f}$ decreased with increasing fiber content, which is ascribed to the fiber cluster in the matrix. Approximately 30% of the fibers showed orientation angles between 45° and 50°, while most of the fibers (approximately 87%~90%) ranged from 25° to 65° according to the analysis of the orientation angles.

(4) When the fiber content was lower than 2.0 wt.%, the distribution and orientation of the carbon fibers showed no apparent effect on the electrical resistivity. As the fiber content exceeded 2.0 wt.%, a conductive network of carbon fibers was formed, and the electrical resistivity was further reduced. However, excessive carbon fiber content would cause fiber clusters, leading to a decreasing uniformity of the fiber dispersion. A quantitative relationship between the distribution coefficient (*α_f_*) and electrical resistivity ($\rho_{c}$) was obtained based on the Taipalus model, and the expression was $\alpha_{f}=0.9236-448.0652/\rho_{c}$, with an R-square value of 0.976. This formula can be used to predict the dispersion of carbon fibers with contents higher than 2 wt.% by measuring the electrical resistivity.

(5) The flexural strength ($\sigma_{cft})$ of the contact-hardening composites was dependent on the effective carbon fiber numbers, the product of the total number of the fibers ($X_{total})$ in the composites and the distribution coefficient ($\alpha_{f})$. The expression was $\sigma_{cft}=\left(573\alpha_{f}X_{total}+135,155\right)/10,000$.

## Figures and Tables

**Table 1:** Properties of the carbon fibers.

Length (mm)	Diameter (μm)	Density (kg/m^3^)	Aspect Ratio	Tensile Strength (GPa)	Young’s Modulus (GPa)	Electrical Resistivity (Ω·cm)
10	40	1650	2500	1.95	150	290

**Table 2:** Statistical number of carbon fibers in each subsection.

Mix No.	*V_f_* (%)	*X*_1_	*X*_2_	*X*_3_	*X*_4_	*X*_5_	*X*_6_	*X*_7_	*X*_8_	*X*_9_	*X_total_*
1	0.5	61	50	37	22	56	62	9	20	75	392
2	1.0	95	72	134	49	44	76	44	54	73	641
3	2.0	146	142	146	123	96	47	132	151	197	1180
4	3.0	133	440	85	128	89	133	203	148	237	1596
5	4.0	105	71	270	71	317	186	265	387	351	2023

*V_f_*: the mass fraction of carbon fibers.

**Table 3:** Values of the fitting parameters of the model with experimental data.

Fiber Content	*θ* (°)	*cosθ*	*V_p_* (%)	*k*	*b*	${\rho_{\mathrm{model}}}_{\;}(\Omega \cdot \mathrm{cm})$	${\rho_{\mathrm{experiment}}}_{\;}(\Omega \cdot \mathrm{cm})$	Residual (Ω·cm)	*R^2^*
2.0 wt.%	46.2	0.692	2.0	4.91 × 10^−4^	−4.63 × 10^−5^	2480	2550	70	0.976
3.0 wt.%	44.6	0.712	3.0	4.91 × 10^−4^	−4.63 × 10^−5^	1222	1250	28	0.976
4.0 wt.%	45.8	0.697	4.0	4.91 × 10^−4^	−4.63 × 10^−5^	1007	1000	7	0.976

## Data Availability

The data presented in this study are available in the article.

## Sample Availability

Samples of the compounds are available from the authors.
